# PLZF inhibits proliferation and metastasis of gallbladder cancer by regulating IFIT2

**DOI:** 10.1038/s41419-017-0107-3

**Published:** 2018-01-22

**Authors:** Hui Shen, Ming Zhan, Yonglong Zhang, Shuai Huang, Sunwang Xu, Xince Huang, Min He, Yanhua Yao, Mohan Man, Jian Wang

**Affiliations:** 1 Department of Biliary-Pancreatic Surgery, Renji Hospital, School of Medicine, Shanghai Jiao Tong University, 1630 Dongfang Road, Shanghai, 200127 China; 2 Department of Biochemistry and Molecular Cell Biology, Shanghai Key Laboratory of Tumor Microenvironment and Inflammation, Institutes of Medical Sciences, School of Medicine, Shanghai Jiao Tong University, Shanghai, 200025 China

## Abstract

Gallbladder cancer (GBC) is a malignant cancer with very poor prognosis. Although promyelocytic leukemia zinc-finger protein (PLZF) was reported to be deregulated in numerous cancers and also relevant to clinical prognosis, its role in GBC progression has been little known. In this study, we found PLZF expression was decreased in GBC, correlating to advanced TNM stage, distant metastasis, and shorter overall survival. Moreover, ectopic PLZF expression in GBC cells (NOZ and GBC-SD) significantly reduced the cell proliferation, migration, and invasion. Consistently, overexpression of PLZF in xenograft mice model could suppress tumor growth and liver metastasis. Mechanical investigations verified PLZF could regulate the expression of cell cycle arrest-associated gene p21 and epithelial–mesenchymal transition (EMT)-related genes (E-cadherin and N-cadherin) in GBC cell lines. Importantly, PLZF remarkably increased the mRNA transcription of interferon-induced protein with tetratricopeptide repeat 2 (IFIT2) by increasing STAT1 protein level, a known factor involved in tumor progression. Furthermore, ablation of IFIT2 in PLZF overexpression cells abrogated the tumor-suppressive function of PLZF, at least partially, leading to impaired tumor growth and EMT program. These studies indicated PLZF inhibited the proliferation and metastasis via regulation of IFIT2. In conclusion, our study demonstrated PLZF could be a promising tumor biomarker for GBC, and also be a potential therapeutic target.

## Introduction

Gallbladder cancer (GBC) is a highly lethal and the most common biliary tract cancer, ranking the sixth leading cause of cancer-related death of digestive system^1,2^. Due to easier local infiltration and distant metastasis, GBC has an extremely poor prognosis with the median survival of 9.2 months and the 5–year survival rate of 5%^3,4^. Given the lack of specific tumor marker and effective therapeutic targets for GBC, novel insight into the GBC progression contributes to the identification of potential targets for early diagnosis and therapy.

One protein of Kruppel-like zinc-finger proteins family, promyelocytic leukemia zinc-finger protein (PLZF), also known as ZBTB16, was first discovered in acute promyelocytic leukemia as a fusion protein with the retinoic acid receptor α^5,6^. PLZF is involved in diverse cellular processes, particularly in stem cells self-renewal or differentiation, and immune cells development^7^. Nevertheless, the role of PLZF appeared to be controversial in tumor progression. Several studies showed PLZF was able to reduce cell growth and survival in numerous cancers including melanoma, malignant mesothelioma, prostate cancer, and non-small cell lung cancer cells via c-myc suppression or poly ADP-ribose polymerase (PARP) and Mcl-1 expression increase^8–13^. Importantly, low expression of cytoplasmic PLZF strongly correlated with high tumor grade, lymph node metastasis and indicated a short overall survival (OS) time in non-small cell lung cancer^14^. Meanwhile, Hur et al. claimed a tumor-promoting effect of PLZF by repressing the p53 pathway^15^. In thyroid carcinoma, high cytoplasmic expression of PLZF was found to be involved in capsular invasion and lymph node metastasis^16^. However, the role of PLZF in GBC has remained to be elucidated.

Interferon-induced protein with tetratricopeptide repeat 2 (IFIT2) is a member of IFN-stimulated genes (ISGs), which are induced after the treatment of type I or III IFNs^17^. It could constitute complexes with itself or with two other related human ISGs, IFIT1 and IFIT3. In addition, IFIT2 has been considered as a tumor suppressor in many tumors to promote cellular apoptosis, suppress tumor proliferation, and metastasis^18^.

In the present study, we provided evidences in vitro* and* in vivo that PLZF served as a potent tumor suppressor for decreased tumor growth and liver metastasis of GBC. Moreover, IFIT2 expression has been markedly increased following PLZF overexpression, and was demonstrated to be required for the tumor inhibition of PLZF in GBC cells. Therefore, our study demonstrated PLZF reduced GBC progression by IFIT2-dependent p21 increase and suppression of tumor epithelial–mesenchymal transition (EMT). Also, we found PLZF promoted the transcription of IFIT2 by increasing STAT1 protein level. Hence, our study provided a valuable biomarker for prognosis and a potential therapeutic target for GBC.

## Materials and methods

### Tissue samples

Formalin-fixed, paraffin-embedded (FFPE) tumor samples with histologically confirmed GBC were obtained from 80 patients who had GBC surgical resection and postoperative adjuvant chemotherapy at the Department of Pathology (Renji Hospital) from January 2004 to February 2015. Twenty FFPE gallbladder samples were obtained from gallbladder stone patients. Matched fresh primary GBC samples and relevant non-tumorous tissues were obtained from 15 patients among the 80 GBC patients. All the fresh specimens were routinely snap-frozen in liquid nitrogen. The paraffin-embedded samples for immunohistochemistry (IHC) were evaluated by two certified pathologists at the Department of Pathology. Follow-up data and medical records were collected from the hospital electronic medical records. And the acquisition of patient specimens and medical data got the permission from Ethical Committee of Renji Hospital, Shang Hai Jiao Tong University School of Medicine and the patients or their relatives.

### Cell lines

The human embryonic kidney 293T cells and human GBC cell line GBC-SD were purchased from the Chinese Academy of Life Sciences (Shanghai, China). Another GBC cell line NOZ was kindly gifted by Xinhua hospital (Shanghai, China).

GBC-SD and 293T cells were maintained in RPMI-1640 medium (GibcoBRL, Gaitherburg, MD, USA). NOZ cells were maintained in Willian’s E medium. All the cells were maintained in the medium supplemented with 1% antibiotics and 10% fetal bovine serum (GIBCO) at 37° C with 5% CO_2_.

### Plasmids construction, transfection, and lentiviral transduction

Human PLZF expression plasmid was constructed by insertion of the coding sequence (CDS) of PLZF into pCDH-CMV plasmid (System Biosciences, CA, USA). STAT1 plasmid was kindly gifted by Hao Jia from Department of Biochemistry and Molecular Cell Biology, Shanghai Jiaotong University School of Medicine. The PLZF short hairpin RNA (shRNA) was constructed by Shanghai GenePharma Medical Biotechnology Company. The IFIT2 shRNA was constructed by Shanghai GenePharma Medical Biotechnology Company. PCDH-flag-PLZF sense: 5′-TGCTCTAGAGCAATGGATTACAAGGATGACGACGATAAGGATCTGACAAAAATGGGCAT GATC-3′; PCDH-flag-PLZF antisense: 5′-CCGGAATTCCGGTCACACATAGCACAGGTAGAGGTACG-3′. The sense sequence of IFIT2 shRNA was: 5′-CCAAATCCTTCATGTAATA-3′. The sequence of the IFIT2 shRNA was the following: 5′-TGCTGTTGACAGTGAGCGCGCCAAATCCTTCATGTAATATTAGTGAAGCCACAGATGTAATATTACATGAAGGATTTGGCTTGCCTACTG CCTCGGA-3′. Recombinant lentiviruses were produced using HEK-293T cells and the viruses were harvested and used to infect human GBC cells with 4 μg/ml polybrene (Sigma). Then, the stable expression cells were selected by 2.5 μg/ml puromycin. Mock were these empty vector stably transfected cells and NC were these negative control plasmid stably transfected cells.

### RNA extraction and miRNA analysis

Total RNA was isolated from cell lines using Trizol reagent (Invitrogen) according to the manufacturer’s instructions. Complementary DNAs were synthesized using Reverse Transcriptase kit. The gene expression levels were detected by using SYBR Premix Ex Taq (Takara, Shiga, Japan) and performed real-time PCR on the ABI Prism 7500 system (Applied Biosystems, Foster City, CA). Data were normalized to internal control β-actin.

The primers were PLZF-forward: 5′-TGCGGCTGAGAATGCATTA-3′; PLZF-reverse: 5′-ACACAGCAGACAGAAGACGG-3′. IFIT2-forward: 5′-CTCAAAGGGCAAAACGAGGC-3′; IFIT2-reverse: 5′-CCAGGCATAGTTTCCCCAGG-3′. β-Actin-forward: 5′-GGACTTCGAGCAAGAGATGG-3′; β-actin-reverse: 5′-AGCACTGTGTTGGCGTACAG-3′. All the primers above were provided by Biosune Biotech. The experiments were done in triplicates.

### Co-immunoprecipitation (co-IP), protein extraction, and western blot (WB) analysis

Plasmids encoding STAT1 and PLZF-Flag proteins were transiently transfected into HEK-293T cells, and 48 h after transfection, cells were lysed in IP lysis buffer with protease inhibitor cocktail and phenylmethanesulfonyl fluoride (PMSF). Co-IP assays were performed with Flag beeds (Sigma-Aldrich; M8823). For normal western blot, total protein was extracted from GBC cells using RIPA lysis buffer supplemented with 1% PMSF and proteinase inhibitor cocktail. Bicinchoninic acid (BCA) assay was used to measure the protein concentration. Equal amounts of protein were loaded on a 8% sodium salt -polyacrylamide gel electrophoresis (SDS-PAGE) and transferred to NC membranes (Millipore, Bedford, MA). Then, the blots were blocked in 5% skimmed milk with 0.1% Tween 20 for 1 h at room temperature followed by incubating at 4° C overnight with primary antibodies. The membranes were washed with tris-buffered saline and tween (TBST) and then incubated with secondary antibody at room temperature for 2 h. The blots were detected by ECL chemiluminescence kit (Millipore). The PLZF antibody was obtained from Santa Cruz Biotechnology (Santa Cruz, CA). β-Actin antibody from Abclonal Biotech was used as loading control. Other antibodies were purchased from Proteintech group (Proteintech, USA).

### Luciferase gene report assay

The plasmid containing the IFIT2 promoter region was kindly gifted by Hao Jia from Department of Biochemistry and Molecular Cell Biology, Shanghai Jiaotong University School of Medicine. The IFIT2 promoter and PLZF plasmid were co-transfected into NOZ cells. After 24-h transfection, cells were harvested and the luciferase activities were measured by the Dual-Luciferase Reporter Assay System (Promega). The values of luciferase assay were expressed after normalization to Renilla luciferase activity. Each transfection was carried out in triplicates.

### Immunofluorescence

We also performed immunofluorescence (IF) staining to detect the function of PLZF on IFIT2. In all, 2 × 10^4^ PLZF overexpression cells were seeded to 24-well plate covered with sterile coverslips. The cells were incubated for another 12 h. Then, the primary antibody and matched secondary antibody were adopted to stain PLZF and IFIT2. In all, 4, 6-diamidino-2-phenylindole (DAPI) was used to stain cell nuclei. All assays were performed in triplicates.

### CCK-8 and plate colony formation assays

Cell Counting Kit-8 (CCK-8) and plate colony formation assays were performed to detect the cell proliferation capacity. For CCK-8 assay, 2000 cells with 100 μl medium were seeded into each well of 96-well plates. Considering of the evaporation, periphery wells of 96-well plates did not seed cells. After every 24 h, 10 μl CCK-8 was added to the medium for 1 h at 37° C and then we measured the absorbance of the plates at 450 nm. For the plate colony formation assay, 600 cells with 2.5 ml medium were seeded into each well of six-well plates and then maintained at 37° C with 5% CO_2_. After 2-week growth, the cells were fixed and stained with Coomassie Brilliant Blue for 30 min. The number of clones was counted and all the assays were repeated three times.

### Cell migration and invasion assays

Transwell assay was used to assess the migration and invasion abilities of the GBC cells. Twenty-four-well transwell chambers with 8 μm pore size polycarbonate membrane (Corning, NY, USA) were used in this assay. For migration assay, 4 × 10^4^ cells with 100 μl serum-free medium were seeded into the upper chamber. In the lower chamber, 700 μl medium with 10% fetal bovine serum was added. After 16 h, the migrated cells were fixed with 4% paraformaldehyde and stained with Coomassie Brilliant Blue. The cells were stained with Coomassie Brilliant Blue. Then, the cells were counted in six random fields of each chamber. For invasion assay, 8 × 10^4^ cells were seeded into the upper chamber with Matrigel (BD)-coated membrane for 48 h. All experiments were carried out in triplicates.

### Immunohistochemistry

Specimens fixed in 10 % buffered formalin were made into paraffin-embedded sections. Sections were deparaffinized and rehydrated by dimethylbenzene and ethanol. The antigen activity of specimens was retrieved by 0.01 M sodium citrate buffer (pH 6.0). Later, the sections were kept in 3% hydrogen peroxide for 20 min to erase endogenous peroxidase. Then, the sides were blocked with goat serum and incubated with primary and secondary antibody. 3, 3'-diaminobenzidine (DAB) system was applied in the positive staining. The final results were assessed independently by two pathologists who knew nothing about the group design. The scoring of the staining was based on the intensity and proportion of the positive staining. The staining intensity was classified into four groups (0 = negative, 1 = weak, 2 = moderate, 3 = strong); and for the staining area (0 = negative, 1 = 1–9 %, 2 = 10–39 %, 3 = 40–69 %, and 4 = 70–100 %).

### In vivo studies

To investigate the proliferation effect of PLZF in vivo, 100 μl phosphate-buffered saline (PBS) containing 1 × 10^6^ NOZ cells were injected subcutaneously into the right lower regions of 4-week-old male nude mice. Tumor length (L) and width (W) were measured every 3 days with vernier caliper. The tumor volume (V) was calculated by the following formula: V = L × W^2^ × 0.5^19^. After 5 weeks, all the mice were sacrificed and the subcutaneous xenografts were excised. To further verify the metastatic capability of PLZF in vivo, 100 μl PBS containing 5 × 10^6^ NOZ cells were injected subcutaneously into the nude mice. After 7 weeks from the injection, the mice were sacrificed and the livers were excised and made into sections for hematoxylin and eosin (H&E) staining and IHC staining. All the animal experiments were approved by the Institutional Review Board of the Renji Hospital of Shanghai Jiao Tong University.

### Statistical analysis

All data were presented as the mean ± s.d. The variance of each experimental group was determined by an unpaired two-tailed Student’s *t*-test. Clinicopathological data were analyzed using Pearson’s Χ^2^ test. Kaplan–Meier method and log-rank test were performed to analyze the survival probabilities. Univariate and multivariate analysis were analyzed using Cox proportional hazard regression model. All of the statistical analyses were performed with SPSS 19.0 software. *P*-values <0.05 were considered statistically significant.

## Results

### PLZF expression was significantly decreased in GBC and low PLZF expression predicted poor clinical outcome

**Fig. 1:**
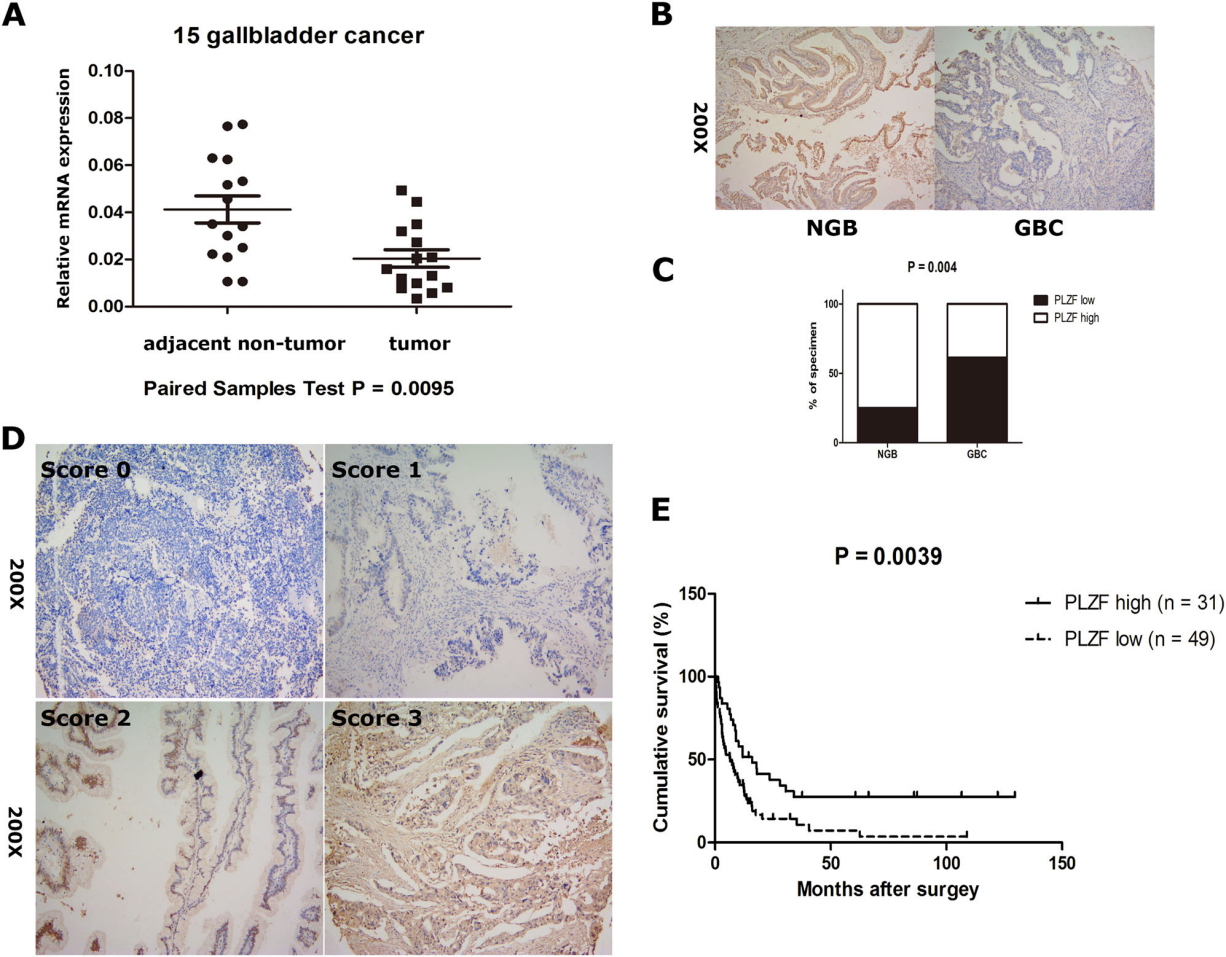
PLZF was downregulated in GBC and correlated with overall survival **a** qRT-PCR showed relative PLZF mRNA level was significantly lower in GBC tissues than those in adjacent non-neoplastic tissues. **b** Representative IHC staining images of normal gallbladder (NGB) and GBC tissues. **c** Quantification of PLZF expression according to IHC scores (see below) in NGB (n = 20) and GBC (n = 40). **d** Representative IHC staining images of different scores, which were calculated by intensity and percentage of stained cells as described in the methods. **e** GBC patient survival was analyzed by Kaplan–Meier analysis. *P*-value was calculated using log-rank test. Results showed lower expression of PLZF correlated with poorer prognosis

In 15 pairs of GBC tissue samples, the mRNA expression levels of PLZF were detected to be lower in GBC tissues compared with that in adjacent non-neoplastic tissues (Fig. 1a). Then, we examined PLZF protein level by performing IHC staining assay in 20 normal gallbladder (NGB) tissues and a tissue microarray including 80 GBC samples. As shown in Fig. 1b, the positive staining of PLZF was mainly observed in the cytoplasm. Compared with NGB tissues, PLZF was significantly decreased in GBC (Fig. 1b). Among the 80 cases of GBC specimens, 61% (49/80) of cases showed lower expression and 39% (31/80) showed higher expression; whereas in the NGB tissues, 5 (25%) exhibited lower and 15 (75%) exhibited higher expression (Fig. 1c). To further investigate the clinical significance of PLZF expression in GBC, we evaluated the correlation between PLZF expression and the clinicopathological characteristics through statistical analysis. Patients were classified into PLZF low and PLZF high groups based on the staining intensity of median expression level of PLZF (PLZF low, score 0,1; PLZF high, score 2,3, Fig. 1d, described in Materials and methods section). The clinicopathological characteristics of the 80 patients were described in Table 1. We found that lower expression of PLZF was correlated with advanced Tumor Node Metastasis (TNM) stage (*P* = 0.012) and distant metastasis (*P* = 0.008).

**Table 1:** Correlation of PLZF expression with the clinicopathological features of GBC

	PLZF (high)		PLZF (low)		*P*-value
	*N* = 31	%	*N* = 49	%	
Sexual					0.249
Male	7	8.8	17	21.2	
Female	24	30.0	32	40.0	
Age (years)					0.702
<65	14	17.5	20	25.0	
≥65	17	21.3	29	36.2	
Tumor size (cm)					0.444
<4	16	20.0	21	26.2	
≥4	15	18.8	28	35.0	
Differential grade					0.851
I–II	19	23.8	29	36.2	
III–IV	12	15.0	20	25.0	
TNM stage					0.012*
I–II	19	23.8	16	20.0	
III–IV	12	15.0	33	41.2	
Lymph node metastasis					0.102
No	26	32.5	33	41.2	
Yes	5	6.3	16	20.0	
Distant metastasis					0.008*
No	25	31.3	25	31.2	
Yes	6	7.5	24	30.0	

χ^2^ test was performed

*P*  <  0.05 was considered statistically significant, * P < 0.05.

Kaplan–Meier survival analysis combined with the log-rank test for OS was performed to evaluate the correlation between low PLZF expression and prognosis (Fig. 1e, Table 2), and indicated low expression level of PLZF patients was associated with shorter OS than those with higher PLZF expression (hazard ratio (HR), 0.47; 95% confidence interval (CI), 0.28–0.80, *P* = 0.005). Moreover, tumor size (HR, 1.71; 95% CI, 1.05–2.82, *P* = 0.033), TNM stage (HR, 2.27; 95% CI, 1.36–3.77, *P* = 0.002), lymph node metastasis (HR, 2.16; 95% CI,1.25–3.72, *P* = 0.006), and distant metastasis (HR, 2.36; 95% CI, 1.42–3.91, *P* = 0.001) were also risk factors for OS. Besides, multivariate analysis using Cox model, including tumor size, TNM stage, lymph node metastasis, distant metastasis, and PLZF expression showed that only PLZF was an independent prognostic factor for OS in GBC patients (HR, 0.48; 95% CI, 0.28–0.83, *P* = 0.008, Table 2). The results suggested patients conducting PLZF low expression tended to have a poor prognosis.

**Table 2:** Univariate and multivariate analysis of the correlation of prognosis with PLZF and clinicopathologic data in GBC

Variable	Univariable analysis		Multivariable analysis	
	HR (95% CI)	*P*-value	HR (95% CI)	*P*-value
Sex (male vs. female)	1.13 (0.67–1.92)	0.646		
Age (<65 vs. ≥65)	1.24 (0.76–2.02)	0.39		
Histological grade (III–IV vs. I–II)	1.18 (0.72–1.94)	0.501		
Tumor size (≥4 vs. <4)	1.71 (1.05–2.82)	0.033*	1.32 (0.75–2.33)	0.328
TNM stage (III–IV vs. I–II)	2.27 (1.36–3.77)	0.002*	1.47 (0.73–2.94)	0.281
Lymph node metastasis (presence vs. absence)	2.16 (1.25–3.72)	0.006*	1.41 (0.68–2.94)	0.359
Distant metastasis (presence vs. absence)	2.36 (1.42–3.91)	0.001*	1.20 (0.55–2.63)	0.648
PLZF expression (high vs. low)	0.47 (0.28–0.80)	0.005*	0.48 (0.28–0.83)	0.008*

*HR* hazard ratio; *CI* confidence interval

*P*  <  0.05 was considered statistically significant, * P < 0.05.

### PLZF overexpression reduced GBC cells proliferation

**Fig. 2:**
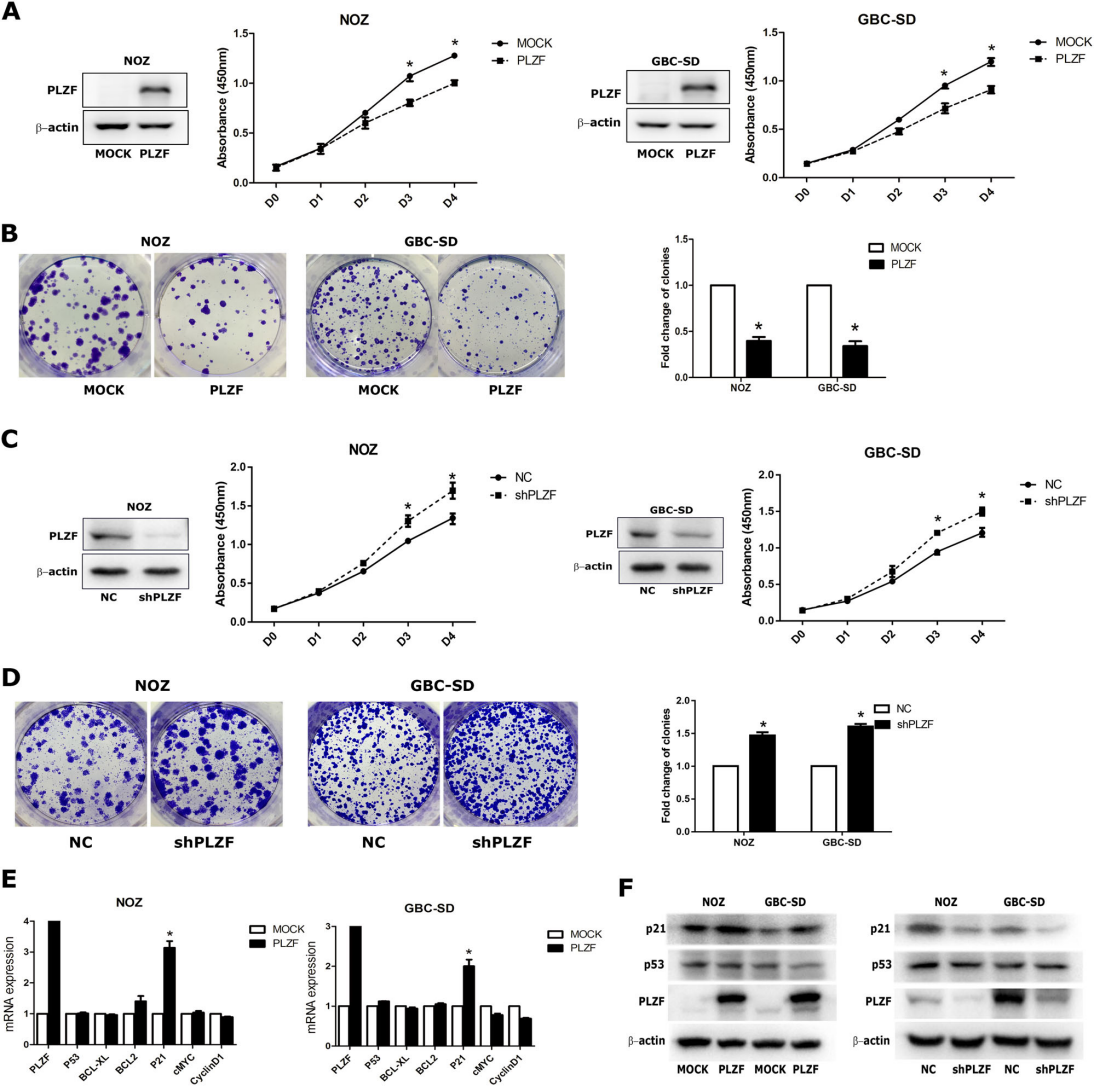
PLZF suppressed GBC cell proliferation **a** PLZF expression in NOZ and GBC-SD cells stably expressing MOCK or PLZF were analyzed by western blot and CCK-8 assay was shown. **b** Colony-forming assay was performed in NOZ and GBC-SD cells stably expressing MOCK or PLZF. Relative clone numbers are shown. **c** PLZF expression in NC and shPLZF cells were analyzed by western blot and CCK-8 assay was shown. **d** Colony-forming assay was performed in NC and shPLZF cells. **e** Expression of genes involved in proliferation and apoptosis were detected by qRT-PCR in MOCK and PLZF-overexpressing cells. **f** The protein levels of p21 and p53 were detected by western blot. (* P < 0.05)

To identify the effect of PLZF on the proliferation of GBC cells, we performed CCK-8 and colony formation assay in NOZ and GBC-SD cells stably overexpressing PLZF. As shown in Fig. 2a, PLZF-overexpressing cells displayed slower proliferation activity. Furthermore, PLZF reduced the colony formation rates, indicating attenuated colony formation ability. Smaller colonies were also observed in PLZF overexpression cells compared with the MOCK cells, which stably transfected with empty vector (Fig. 2b). Meanwhile, knockdown PLZF expression could significantly enhance the proliferation of GBC cells (Figs. 2c, d) compared with NC group, which stably transfected with negative control plasmid. We also detected whether PLZF could affect the apoptosis of GBC cells, but the apoptosis analysis by flow cytometry results showed no big difference between MOCK and PLZF overexpression cells (Supplement 1). As PLZF has been shown to regulate the downstream genes mainly via binding to the genes’ promoter^7^, we performed quantitative real-time PCR (qRT-PCR) to detect the expression changes of genes correlating with cell proliferation and apoptosis, and found p21 was significantly upregulated both in overexpressing PLZF NOZ and GBC-SD cells (Fig. 2e). p21 is mainly regulated by p53, then we detected the expression of p53 both in mRNA and protein level. The results showed PLZF effect on p21 was independent of p53 (Fig. 2f). As p21 is an important tumor-suppressor gene, PLZF may suppress the proliferation of GBC cells by increasing p21 expression. Collectively, these observations indicated PLZF inhibited GBC cell growth in vitro.

### PLZF inhibited migration and invasion of GBC cells

**Fig. 3:**
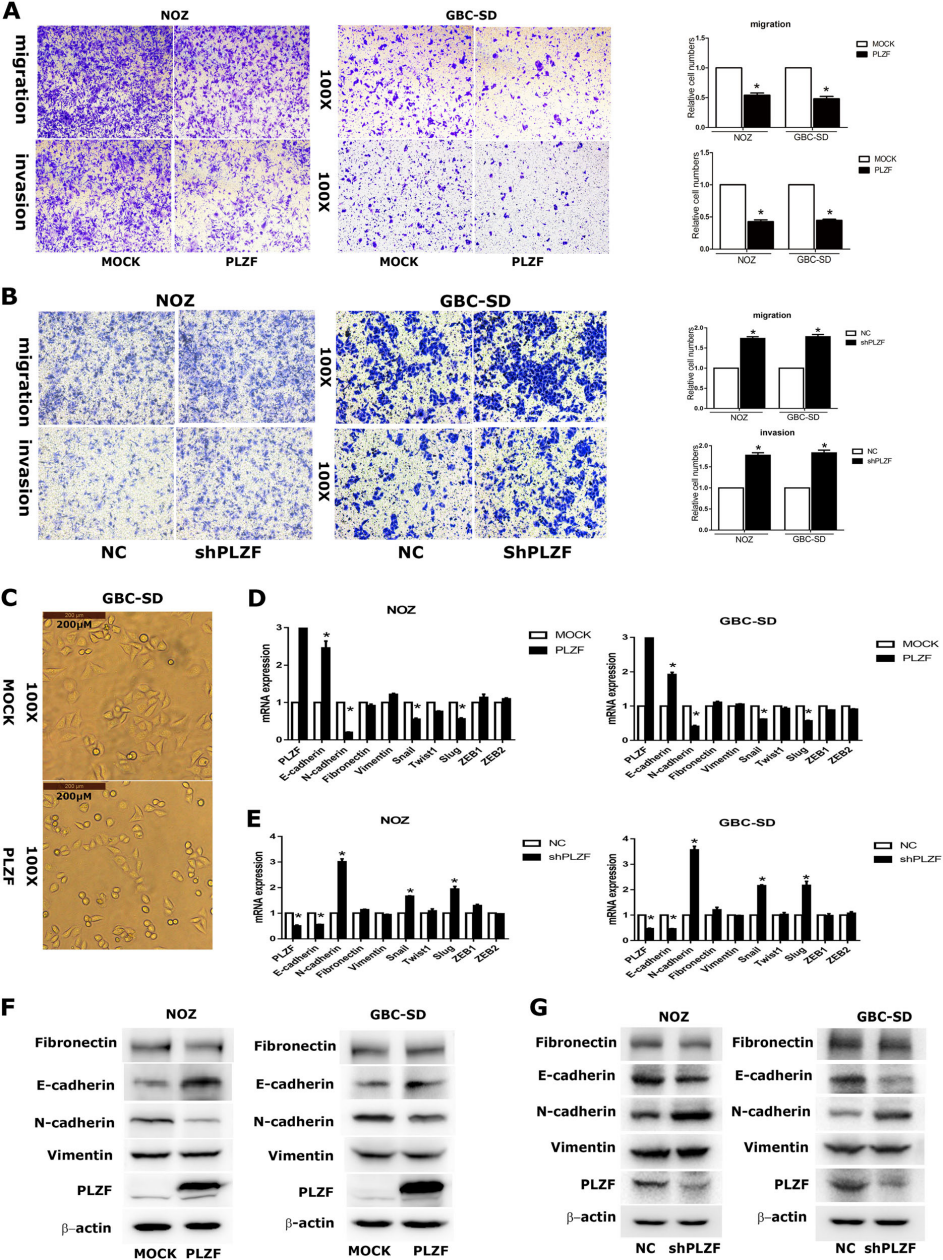
PLZF suppressed GBC cells migration and invasion through EMT **a** Transwell migration and matrigel invasion assays were conducted in cells stably expressing MOCK or PLZF. The relative cell numbers were shown. **b** Transwell migration and matrigel invasion assays were conducted in NC and shPLZF cells. Relative cell numbers are shown. **c** GBC-SD cells underwent morphological change from a rounded or cobblestone-like shape to a spindle shape upon PLZF overexpression. **d** Expression of epithelial marker E-cadherin, mesenchymal markers fibronectin, vimentin, and N-cadherin were detected by qRT-PCR in MOCK and PLZF-overexpressing cells. **e** Expression of EMT markers were detected by qRT-PCR in NC and shPLZF cells. **f** Expression of epithelial marker E-cadherin, mesenchymal markers fibronectin, vimentin, and N-cadherin were detected by western blot in MOCK and PLZF-overexpressing cells. **g** Expression of EMT markers were detected by western blot in NC and shPLZF cells. (P < 0.05)

We next evaluated the migration and invasion ability by transwell migration and matrigel invasion assay. The results showed that the cells on the underside of the filters were significantly decreased in GBC cells overexpressing PLZF compared with mock/vector (Fig. 3a). In addition, we found that PLZF knockdown dramatically enhanced the migration and invasion of GBC cells (Fig. 3b). Moreover, results showed GBC-SD cells underwent morphological change from a rounded or cobblestone-like shape to a spindle shape upon PLZF overexpression (Fig. 3c). As EMT plays crucial role in cancer migration and invasion process, we then detected the mRNA and protein levels of epithelial marker E-cadherin and that of the mesenchymal markers fibronectin, vimentin, and N-cadherin. The qRT-PCR results revealed that E-cadherin gene expression was prominently increased in PLZF overexpression cells, whereas N-cadherin was reduced (Fig. 3d). Inversely, PLZF knockdown could significantly reduce E-cadherin but increase N-cadherin expression (Fig. 3e). The protein levels of E-cadherin and N-cadherin were shown the same variation features (Figs. 3f, g). Furthermore, we investigated the master transcription factors of EMT, such as Snail, Slug, Twist, Zeb1, and Zeb2 by qRT-PCR. The results showed ectopic PLZF expression could suppress Snail and Slug transcription, and PLZF knockdown showed the opposite effect (Figs. 3d, e). The data presented above suggested PLZF could suppress GBC migratory and invasive abilities by preventing EMT program.

### PLZF increased IFIT2 expression by activating STAT1 pathway

**Fig. 4:**
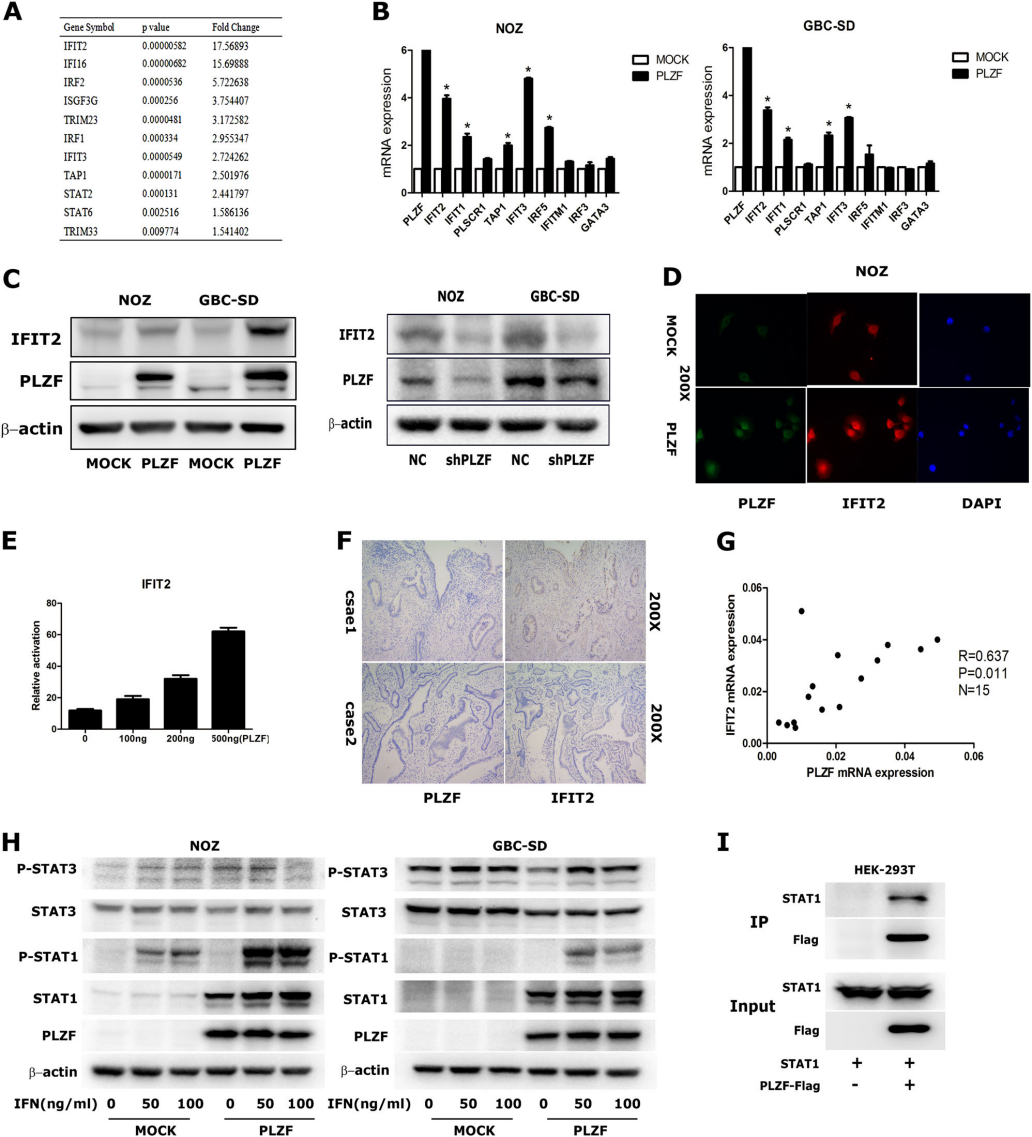
PLZF increased IFIT2 mRNA and protein expression **a** A chip–chip data comparing PLZF-overexpressing cells with control showed PLZF regulated a subset of ISGs. **b** Expression of ISGs were detected by qRT-PCR in NOZ and GBC-SD cells stably expressing MOCK or PLZF. **c** IFIT2 expression was detected by western blot in PLZF-overexpressing and knockdown cells. **d** IFIT2 expression was detected by IF staining in NOZ cells stably expressing MOCK or PLZF. **e** IFIT2 promoter reporter and various amount of PLZF plasmids were co-transfected into NOZ cells. The reporter activity of IFIT2 was measured by luciferase assay. **f** Representative IHC staining images of PLZF and IFIT2 in two GBC cases. **g** Expression of PLZF and IFIT2 in the 15 GBC tissues were positively correlated (R = 0.637, *P* = 0.011). **h** Ectopic expression of PLZF in GBC cells increased STAT1 and phosphorylation of STAT1 when treated with IFNγ. The protein levels of STAT1, pSTAT1, STAT3, and pSTAT3 were detected by western blotting. **i** Co-IP assay using Flag beeds was performed in HEK-293T cells, which transfected with STAT1 and PLZF-Flag plasmids. (P < 0.05)

To explore the underlying mechanism how PLZF suppressed tumor progression, we analyzed a chip–chip data, which compared PLZF overexpression U937T cells with control U937T cells^20^. Interestingly, a subset of ISGs was significantly increased following PLZF overexpression (Fig. 4a). To confirm the regulation of ISGs expression by PLZF, we checked ISGs expression in PLZF overexpression cells by qRT-PCR. As expected, the transcripts of IFIT2, IFIT1, IFIT3, and TAP1 expression levels were increased both in NOZ and GBC-SD cells (Fig. 4b). Among these ISGs, IFIT2 is a well-established tumor suppressor, which was reported to enhance apoptosis, inhibit proliferation, migration, and invasion in numerous cancers. The expression correlation between PLZF and IFIT2 suggested that PLZF effect on GBC proliferation, migration, and invasion might be mediated by regulating IFIT2. We started by verifying IFIT2 protein levels in PLZF overexpression cells. WB and IF staining showed PLZF increased IFIT2 protein levels both in NOZ and GBC cells (Figs. 4c, d). Next, we performed luciferase report assay to evaluate if PLZF might regulate IFIT2 at transcriptional level. The pGL3 vector encoding the region of IFIT2 promoter and different amount of PLZF plasmids were co-transfected into NOZ cells. We found the reporter activity of IFIT2 was proportionally increased in correspondence with the amount of PLZF (Fig. 4e), suggesting PLZF might serve as a transcriptional activator of IFIT2. Moreover, we evaluated the clinical relevance of PLZF and IFIT2 in GBC specimens by IHC staining assay. Like PLZF, IFIT2 exhibited relatively low expression in GBC specimens (Fig. 4f). Seven of 10 GBC tissues were demonstrated weak or negative staining of IFIT2, whereas 30% cases showed IFIT2 high expression. In addition, PLZF and IFIT2 mRNA expression in 15 GBC tissues were detected, which showed PLZF was positively correlated with IFIT2 (Fig. 4g).

To elucidate the molecular mechanism by which PLZF affects IFIT2 expression, we detected the expression of STAT1 and STAT3 by western blotting. Strikingly, total STAT1 and phosphorylation of STAT1 was significantly enhanced in PLZF overexpression cells compared with control cells after the stimulation of IFNγ, but there was no change of total STAT3 and phosphorylation of STAT3 (Fig. 4h). Also, we detected the mRNA levels of STAT1α and STAT1β following PLZF overexpression or PLZF downregulation by qRT-PCR and the results showed no difference (Supplement 2). Then, we performed co-IP assay in HEK-293T cells, which transfected with STAT1 and PLZF-Flag plasmids and results showed STAT1 could interacted with PLZF protein (Fig. 4i). These results, taken together, indicated that PLZF promoted the transcription of IFIT2 by increasing STAT1 protein level.

### PLZF suppressed the proliferation and invasion of GBC cells via IFIT2

**Fig. 5:**
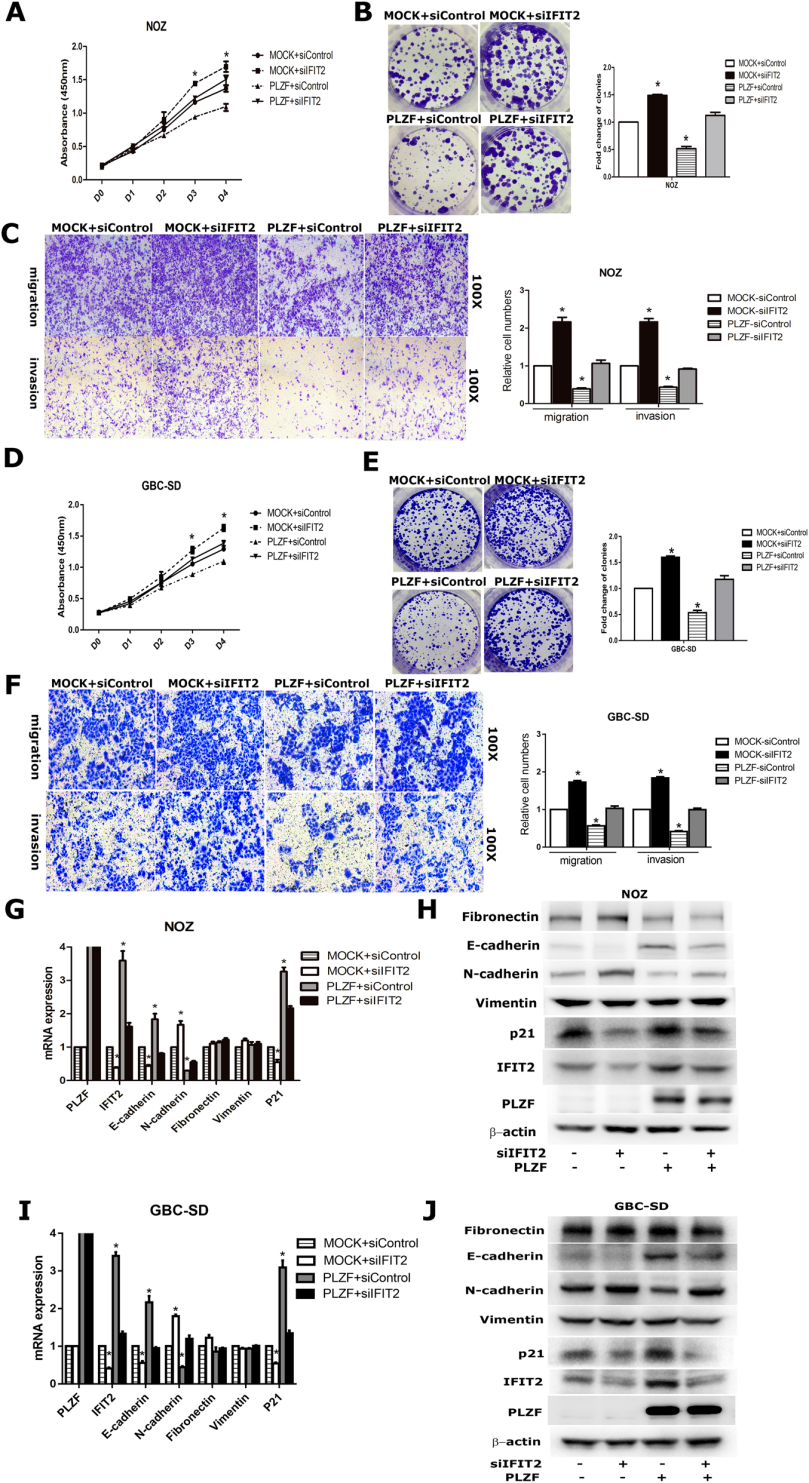
IFIT2 knockdown reversed the function of PLZF in NOZ cells **a**, **d** CCK-8 assay was conducted in NOZ and GBC-SD cells transfected with MOCK + siControl, MOCK + siIFIT2, PLZF + siControl and PLZF + siIFIT2. **b**, **e** Colony formation assay was conducted in NOZ and GBC-SD cells transfected with MOCK + siControl, MOCK + siIFIT2, PLZF + siControl and PLZF + siIFIT2. Relative clone numbers are shown. **c**, **f** Transwell migration and matrigel invasion assays were conducted in NOZ cells transfected with MOCK + siControl, MOCK + siIFIT2, PLZF + siControl and PLZF + siIFIT2. Relative cell numbers are shown. **g**, **i** Expression of EMT markers and p21 were detected by qRT-PCR in GBC cells transfected with MOCK + siControl, MOCK + siIFIT2, PLZF + siControl and PLZF + siIFIT2. **h**, **j** Expression of EMT markers and p21 were detected by western blot. (P < 0.05)

As we have verified PLZF could regulate the expression of IFIT2, we assumed that the function of PLZF might be dependent on IFIT2. Hence, we depleted IFIT2 expression by siRNA-IFIT2 (siIFIT2) transfection in control group and PLZF-overexpressing NOZ cells, followed by examining GBC cell proliferation and migration. Figures 5a, b showed IFIT2 knockdown significantly promoted cell growth and rescued the tumor inhibitory effects following PLZF overexpression. Additionally, evaluation of GBC cell migration and invasiveness capability showed IFIT2 silencing remarkably triggered migration and invasion, whereas the function of PLZF on migration and invasion was abolished by IFIT2 ablation (Fig. 5c). The similar results were obtained in GBC-SD cells (Figs. 4d–f). In accord with the altered phenotypes, IFIT2 depletion rescued the decreased expression of N-cadherin, but suppressed E-cadherin and p21 expression (Figs. 5g–j). Taken together, the results suggested PLZF inhibited proliferation, migration, and invasion through IFIT2 pathway.

### PLZF was essential for primary tumor formation and metastasis of GBC

**Fig. 6:**
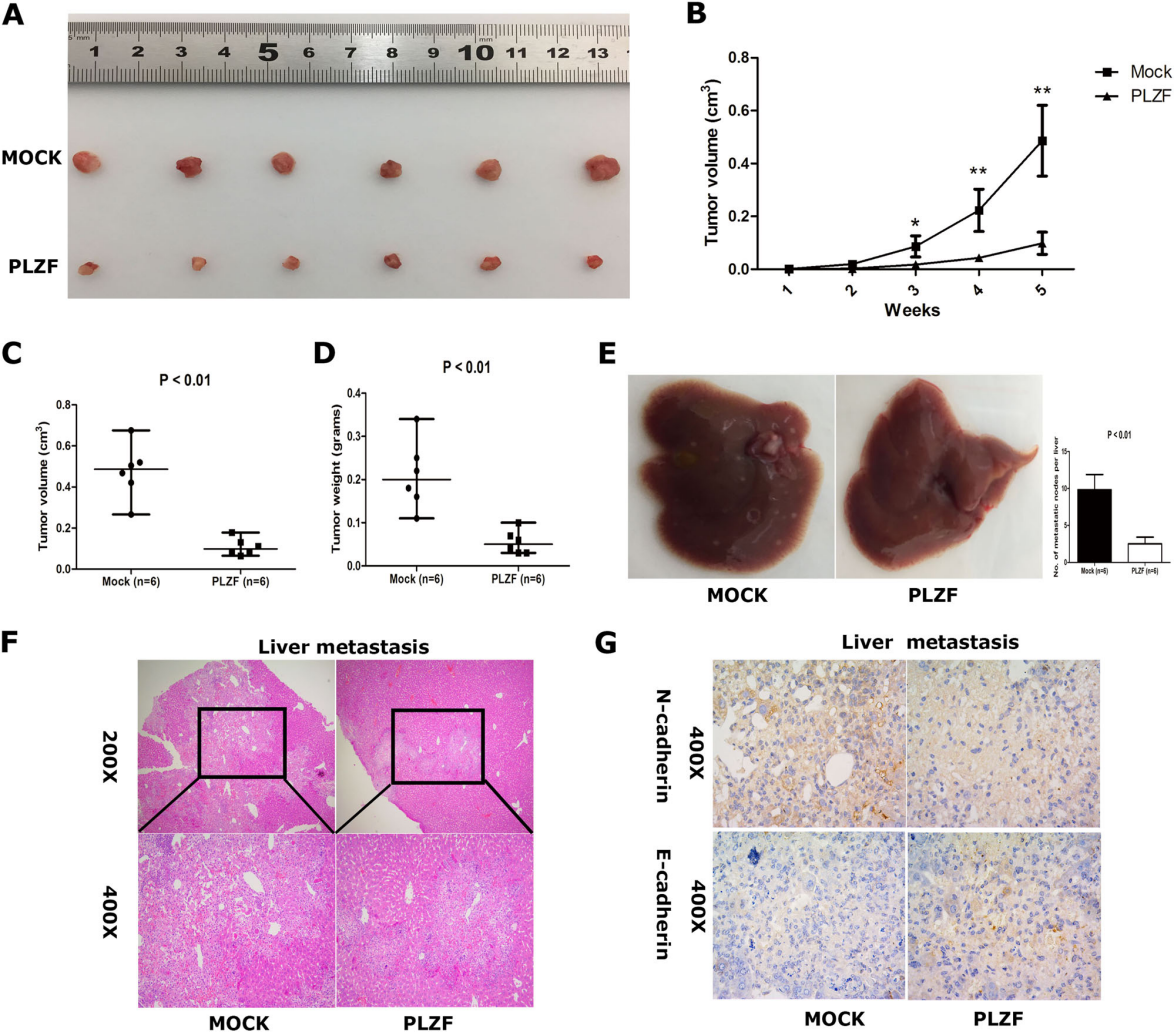
PLZF suppressed tumor growth and metastasis in nude mice **a** NOZ cells stably overexpressing PLZF or vector were injected into the subcutaneous of nude mice. After 5 weeks, subcutaneous xenografts were excised. **b** Tumor weight of each mice injected with NOZ cells stably overexpressing PLZF or vector were measured every week. **c**, **d** Tumor volume and tumor weight were measured. *P*-value was determined by Student's *t*-test. **e** Represent images of mouse livers of Mock or PLZF group are shown and the numbers of metastatic nodes per liver were measured. **f** Represent H&E-staining images of metastatic nodes in mouse livers are shown. **g** Represent IHC staining images of metastatic nodes in mouse livers are shown. (* P < 0.05, ** P < 0.01)

Given that we demonstrated the tumor repressive role of PLZF in vitro, we next determined whether PLZF suppressed GBC cell growth and metastasis in vivo. GBC cells with stable expression of PLZF or control vector were subcutaneously implanted into nude mice. The tumor volumes were measured every 3 days. After 5 weeks, the mice were sacrificed and xenograft tumors were removed and analyzed. As shown in Fig. 6a, the tumors originated from PLZF overexpression cells were remarkably smaller than the control group, whereas PLZF overexpression group had smaller tumor volume and tumor weight (Figs. 6b–d). For metastasis assay, after approximately 7 weeks post injection, the mice organs were harvested for H&E analysis. Multiple lesions in livers were observed in control mice. Surprisingly, a few nodes were observed in mice livers ectopically expressing PLZF (Fig. 6e). H&E staining confirmed less metastatic nodes in mice livers of PLZF overexpression compared with the control group (Fig. 6f). Importantly, we also found PLZF-overexpressing group had low expression of N-cadherin and high expression of E-cadherin compared with control group (Fig. 6g). The IHC staining of liver metastatic nodes from nude mice further proved PLZF regulated EMT. In summary, PLZF suppressed tumor growth and metastasis in nude mouse model.

## Discussion

GBC is a malignant cancer with very poor prognosis because of its high potential to metastasize in a short time^21^. But the mechanism of tumor proliferation and metastasis is still poorly understood. Cumulative evidences have shown PLZF was downregulated in numerous cancers and relevant with the clinical prognosis. Moreover, the functions of PLZF in cancers remained to be ambiguous and open to interpretations of either a tumor suppressor or tumor promoter as reported previously^7^. However, little is known about the role of PLZF in GBC progression. In this study, the mRNA transcripts of PLZF were significantly downregulated in GBC tissues in contrast to the adjacent counterparts. Consistently, the protein levels of PLZF were reduced in many GBC tissues. Moreover, the decreased PLZF was demonstrated to be associated with tumor size, TNM stage, lymph node metastasis, and distant metastasis. Multivariate analysis using Cox’s regression suggested that PLZF was an independent prognostic factor for OS in GBC patients. In agreement with its role in clinical samples, PLZF was further found to suppress GBC cell growth and metastatic activity in vitro, supporting PLZF being a potential tumor suppressor in GBC.

In this study, we found PLZF overexpression could suppress cell proliferation and tumor growth. Furthermore, we investigated the genes involved in tumor growth and found PLZF could increase p21 mRNA level, but no impact on the apoptotic genes like p53, BCL-2, BCL-XL, and BAX. p21 is a well-studied gene required for cell cycle arrest. Reduced p21 expression was considered as an early event in gallbladder carcinogenesis^22^. Lots of studies have shown p21 has low expression in GBC tissues and absence of p21 expression independently predicts poor outcome^23,24^. Moreover, silence of p21 has been proved to promote GBC cells (NOZ and GBC-SD) proliferation significantly^25^. It is involved in cell proliferation inhibition in both p53-dependent and -independent way^26^. A previous study showed PLZF could interact with some corepressors and bind to p21 promoter to repress its transcription^15^. But our results showed opposite phonotypes. This could be due to the fact that the cells we used were different from those studied or the regulation of PLZF might be context dependent.

EMT is regarded as a potent driver conferring cells with metastatic features, including lower expression of epithelial markers like E-cadherin and higher expression of mesenchymal markers like N-cadherin, vimentin, and Fibronectin^27–30^. EMT gets cells to obtain strong capability of motility and spreading, leading to metastasis to the distant regions^31,32^. The underlying molecular mechanism of PLZF in cancer metastasis remained little understood. Only one study in melanoma demonstrated PLZF suppressed migration and invasion partially through integrin β3 and MMP9^9^. We examined the expression levels of EMT markers for possible mechanism investigations, which showed PLZF overexpression decreased N-cadherin expression but enhanced E-cadherin level, whereas vimentin and fibronectin were not altered, suggesting PLZF could selectively induce mesenchymal markers for initiating EMT program.

To clarify the underlying mechanism of PLZF, we performed chip–chip data analysis using online database and found a link between PLZF and ISGs. Through high-affinity binding with cell surface receptors, IFN molecules can activate the Janus kinase/Signal Transducer and Activator of Transcription (JAK/STAT) signaling pathway, resulting in induction of thousands of ISGs at the transcript level^33–35^. Growing studies have verified ISGs are involved in multiple processes including apoptosis, angiogenesis, and metastasis^33–37^. To validate this prediction, qRT-PCR analysis was used and showed that PLZF overexpression enhanced the expression of a subset of ISGs included IFIT2 in particular. This observation was also confirmed by WB and IF analysis, indicating PLZF could regulate IFIT2 mRNA transcription. Dual-luciferase reporter assays were then performed to further verify PLZF effect on IFIT2 expression regulation. The results showed PLZF increased IFIT2 in a dose-dependent manner. IHC staining of PLZF and IFIT2 in GBC tissues also confirmed a strong correlation. Accumulating evidences have established IFIT2 as a tumor suppressor in various tumor types. IFIT2 promotes apoptosis through the intrinsic apoptosis mechanism by regulating antiapoptotic and proapoptotic factors^17,38–41^. Moreover, IFIT2 was reported to suppress the migration and metastasis by regulating atypical protein kinase C, leading to EMT and cell migration^42^. We therefore verified whether IFIT2 was required for the function of PLZF. We introduced IFIT2 small interfering RNA into PLZF overexpression cells and found the expression of p21 and E-cadherin was decreased, but N-cadherin was increased in PLZF overexpression cells with IFIT2 silencing. Moreover, downregulation IFIT2 expression could rescue the inhibitory effects of proliferation, migration, and invasion in PLZF overexpression cells. IFNs exert their biological effects by activating STATs expression, which translocate into the nuclear and bind ISGs to transactivate their activation^43^. STAT1 is the central mediator of ISGs’ promotion^44^. Notably, we showed that ectopic PLZF expression could enhance STAT1 and pSTAT1 after IFNγ treatment, indicating that PLZF may function as a suppressor of STAT1/IFIT2 pathway. Moreover, our results showed PLZF could interact with STAT1 protein and increase its protein level by an unknow mechanism. Previous studies have demonstrated that STAT1 is mainly degraded by ubiquitination and proteasome pathway^45^. So PLZF might inhibit STAT1 degradation in that way. Collectively, these results suggested PLZF might function concomitantly with IFIT2 for decreased GBC cell proliferation, migration and invasion.

Above all, we revealed that PLZF was remarkably reduced in GBC tissues and low PLZF expression was correlated with advanced TNM stage, distant metastasis, and short overall survival. Furthermore, we found PLZF overexpression inhibited tumor growth and metastasis by activating STAT1/IFIT2 pathway in GBC cells and nude mice. To our knowledge, we are the first to identify the tumor-suppressor role of PLZF in GBC both in vitro* and* in vivo. PLZF might be a potential tumor biomarker and a critical therapeutic target for GBC.

## Electronic supplementary material

Supplement 1

Supplement 2

Supplementary Figure Legends
