# Supplementary figures and images for: PLZF inhibits proliferation and metastasis of gallbladder cancer by regulating IFIT2
Source: Cell Death Dis. 2018 Jan 22;9(2):71. doi: 10.1038/s41419-017-0107-3 (PMC5833736; doi:10.1038/s41419-017-0107-3)

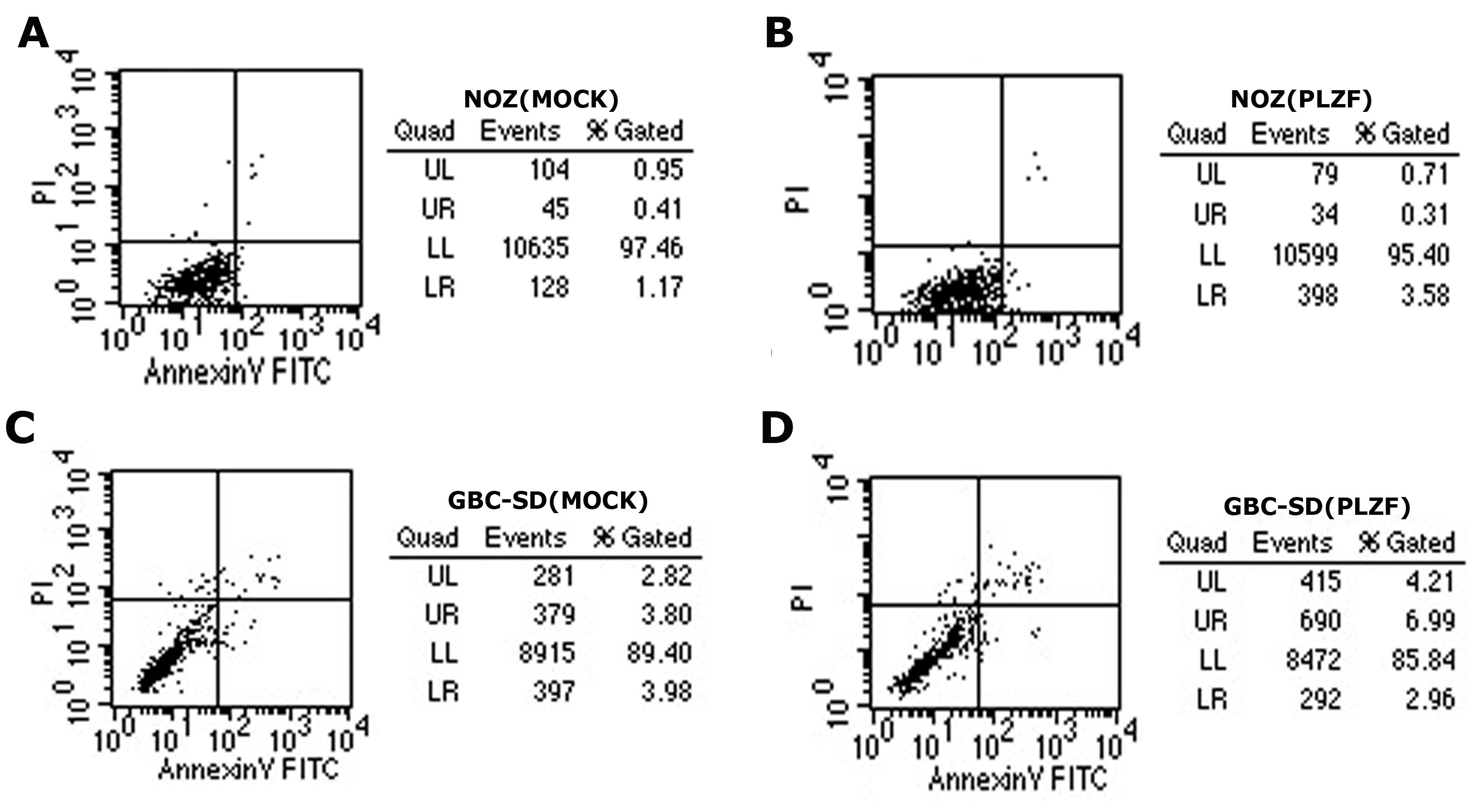

Supplement: Supplementary file 1 — Supplement 1 [file 41419_2017_107_MOESM1_ESM.jpg]

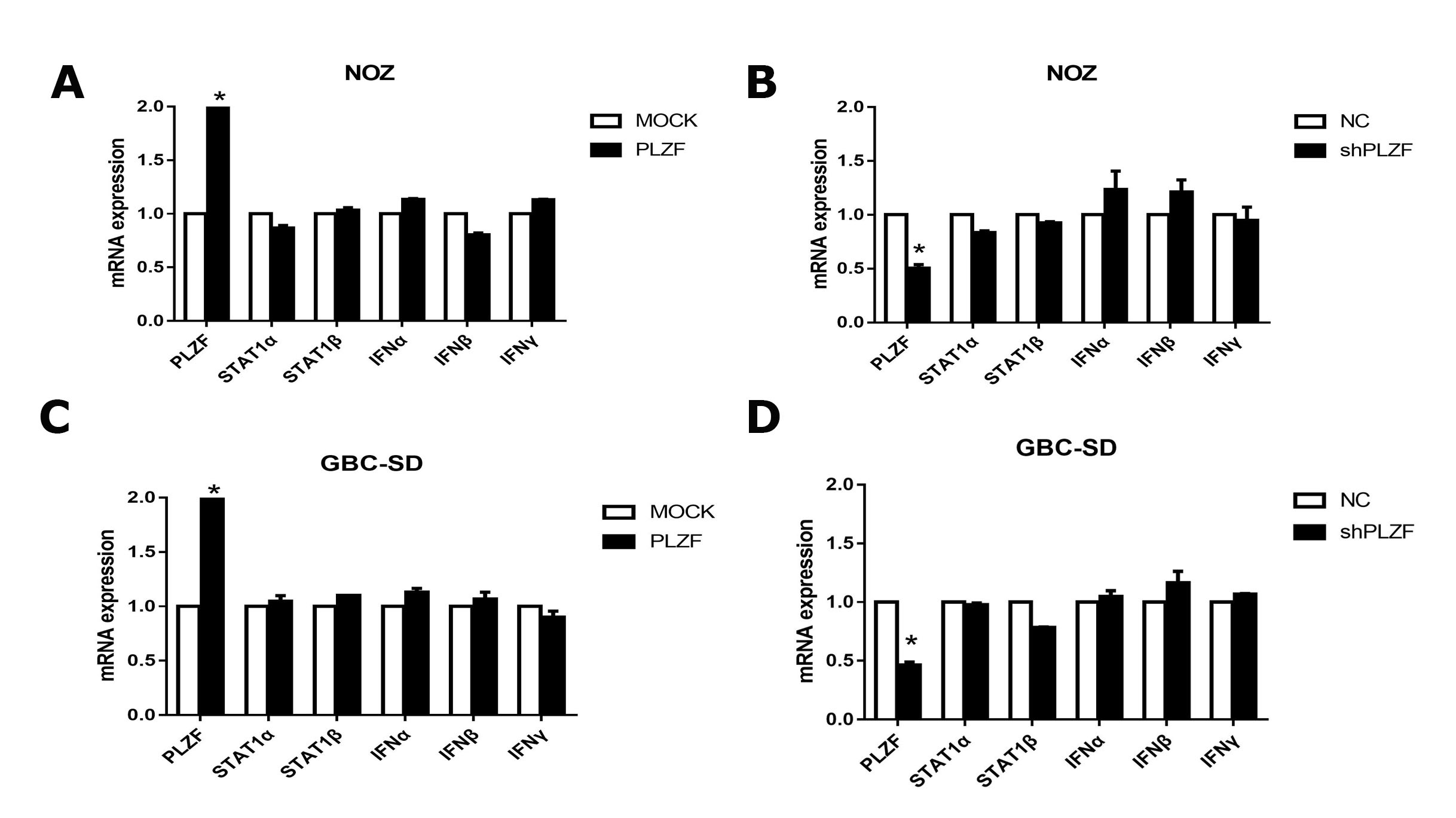

Supplement: Supplementary file 2 — Supplement 2 [file 41419_2017_107_MOESM2_ESM.jpg]
