# Supplementary material for: PLZF inhibits proliferation and metastasis of gallbladder cancer by regulating IFIT2
Source: Cell Death Dis. 2018 Jan 22;9(2):71. doi: 10.1038/s41419-017-0107-3 (PMC5833736; doi:10.1038/s41419-017-0107-3)
Supplement: Supplementary file 3 — Supplementary Figure Legends [file 41419_2017_107_MOESM3_ESM.docx]

**Supplementary Figure Legends**

**Supplementary Figure 1.** Apoptosis analysis by Flow Cytometry results showed no big difference between MOCK and PLZF overexpression GBC cells

**Supplementary Figure 2.** mRNA levels of STAT1α and STAT1β following PLZF overexpression or PLZF downregulation were detected by qRT-PCR and the results showed no difference.
